# GPR68: An Emerging Drug Target in Cancer

**DOI:** 10.3390/ijms20030559

**Published:** 2019-01-28

**Authors:** Shu Z. Wiley, Krishna Sriram, Cristina Salmerón, Paul A. Insel

**Affiliations:** 1 Department of Pharmacology, University of California, San Diego, La Jolla, CA 92093, USA; szwiley@ucsd.edu (S.Z.W.); ksriram@ucsd.edu (K.S.); crsalmeronsalvador@ucsd.edu (C.S.); 2 Department of Medicine, University of California, San Diego, La Jolla, CA 92093, USA

**Keywords:** acidosis, GPR68, proton-sensing GPCRs, RNA-seq, tumor microenvironment

## Abstract

GPR68 (or ovarian cancer G protein-coupled receptor 1, OGR1) is a proton-sensing G-protein-coupled receptor (GPCR) that responds to extracellular acidity and regulates a variety of cellular functions. Acidosis is considered a defining hallmark of the tumor microenvironment (TME). GPR68 expression is highly upregulated in numerous types of cancer. Emerging evidence has revealed that GPR68 may play crucial roles in tumor biology, including tumorigenesis, tumor growth, and metastasis. This review summarizes current knowledge regarding GPR68—its expression, regulation, signaling pathways, physiological roles, and functions it regulates in human cancers (including prostate, colon and pancreatic cancer, melanoma, medulloblastoma, and myelodysplastic syndrome). The findings provide evidence for GPR68 as a potentially novel therapeutic target but in addition, we note challenges in developing drugs that target GPR68.

## 1. Introduction

GPCRs, the largest family of cell signaling receptors (with over 800 GPCRs encoded in the human genome, ~3% of the human genome), are seven transmembrane receptors that respond to numerous types of extracellular signals (e.g., biogenic amines, lipids, peptides, proteins, ions, photons, etc.) and regulate many physiological processes [1]. These include key functional responses, such as neurotransmission, sensory perception, cell metabolism, and differentiation [2]. In addition to modulation of normal physiological functions, GPCRs regulate signal transduction pathways and cellular processes that are critical for initiation and progression of tumors, such as cell proliferation, inhibition of apoptosis, immune evasion, tumor invasion, angiogenesis, and metastasis [3,4]. Upon agonist activation, GPCRs undergo conformational changes and interact with G-protein α, β, and γ subunit heterotrimers. This interaction promotes the exchange of guanosine-5′-triphosphate (GTP) for guanosine-5′-diphosphate (GDP) bound to Gα subunits, leading to dissociation of Gα from Gβγ, both of which can modulate downstream signaling pathways [5]. In addition, activated GPCRs can regulate cell function via β-arrestins, scaffolding proteins for a variety of signaling entities [6].

We recently reported that various human cancers—including CLL (chronic lymphocytic leukemia) cells/stromal cells associated with CLL, colon and breast cancer cell lines, pancreatic ductal adenocarcinoma (PDAC) cells, PDAC tumors, and cancer associated fibroblasts (CAFs)—express from 50 to more than 100 GPCRs [7]. Other work has shown that certain GPCRs and G proteins can undergo activating mutations that enhance the growth of a variety of endocrine tumors and that certain constitutively active GPCRs can be expressed from the genomes of human oncogenic viruses [8,9]. Based on factors that include their accessibility from the extracellular environment, ligand selectivity, expression on particular cell types and tissues, and roles in signal transduction, GPCRs are the molecular entities most commonly targeted by Food and Drug Administration (FDA)-approved drugs, currently serving as drug targets for ~35% of such drugs [10,11]. However, the roles of GPCRs in cancer is under-appreciated compared to that of other signaling entities and currently, only a limited number of drugs used in oncology target GPCRs [9].

Tumors consist of multiple cell types in the TME. In addition to malignant cells, the TME is composed of CAFs (stromal cells), endothelial and other vascular cells, and various types of immune and inflammatory cells. Multiple factors, including insufficient blood perfusion (and hypoxia), inflammation, and glycolytic cell metabolism create an acidic TME [12,13,14]. The physiological pH of blood and tissue is ~pH 7.4 but in the TME the pH can range from 5.5 to 7.0 [12,13].

Acidosis, a defining hallmark of the TME, can regulate proliferation, apoptosis, clonal evolution, and metastasis of cancer cells and modulate inflammation, anti-tumor immunity, and angiogenesis [15,16]. Targeting tumor acidity may aid in the therapy of cancers. Treatment with bicarbonate buffer to increase the pH can reduce growth of melanoma and pancreatic cancer in mice [17], inhibit lung metastases [18], and suppress tumor formation of prostate cancer in genetically engineered mouse models (GEMM) [19]. Several ion channels and receptors help cells respond to extracellular acidosis: acid-sensing ion channels (ASICs), transient receptor potential channel vanilloid subfamily (TRPV), and proton-sensing GPCRs [16]. The proton-sensing GPCR family has four members: GPR4, GPR65 (T lymphocyte death-associated gene 8 protein, TDAG8), GPR68 (OGR1), and GPR132 (G2 accumulation protein, G2A). Upon activation by extracellular acidity, proton-sensing GPCRs activate signaling pathways to regulate tumor biology [15,16]. In this review, we focus on GPR68 and its emerging role in human cancers.

## 2. G Protein-Coupled Receptor 68 (GPR68/OGR1)

### 2.1. Discovery of GPR68/OGR1

GPR68 was first cloned from the HEY human ovarian cancer cell line and named OGR1, although in keeping with revisions in nomenclature, GPR68 is now more commonly used. Fluorescent *in situ* hybridization (FISH) of human lymphocyte chromosomes mapped GPR68 to chromosome 14, band 14q31 [20]. GPR68 has an open reading frame of 1095 nucleotides and encodes a predicted protein of 365 amino acids [20]. Three human mRNA variants of GPR68 have been validated. Each codes for the same GPR68 protein but with differences in the 5′ untranslated region. GPR68 is homologous across several species (e.g., human, mouse, rat, pig, chicken, and zebrafish [21]). Its highest homology is with GPR4: 54% identity from amino acids 13 to 252 and 49% identity from amino acids 258 to 312 [20]. GPR68 was identified as a proton-sensing GPCR, inactive at pH 7.8 but fully activated at pH 6.8, as measured by inositol phosphate (IP) formation [22].

### 2.2. GPR68 Structure

The structure of GPCRs includes an extracellular N-terminal motif followed by seven transmembrane α-helices (I–VII) with three intracellular loops and three extracellular loops, and an intracellular C-terminal domain. Unlike numerous GPCRs, the crystal or cryoelectron microscopic structure of GPR68 has not been resolved. A 3D model [22] has been proposed with a cluster of histidines (H) at the extracellular surface, on top of helices I, IV and VII, and in extracellular loops 1 and 2. In the unprotonated state, helixes I and VII are connected through hydrogen-bond interaction between H20 and H269; a second hydrogen-bond interaction occurs between H17 and H84, which links the N-terminal to extracellular loop 1. Single or double mutations of paired H17-H84 and H20-H269 abolish the proton-sensing function of GPR68 [22].

The mechanism of action of GPR68 is as follows: at slightly alkaline pH, GPR68 is stabilized in an inactive state by hydrogen bonding of the histidines. Protonation of these histidine residues causes loss of hydrogen bonding and presumably repulsion of those residues, allowing the receptor to adopt an active conformation [22]. Zn^2+^ and Cu^2+^ ions are able to coordinate histidine residues and stabilize GPR68 structure in its inactivated conformation; those ions inhibit GPR68-dependent IP formation stimulated at pH 6.9 [22]. An in-frame 450 base pair homozygous deletion in GPR68 deletes four of the seven transmembrane helices and removes three of the six histidine residues thought to be crucial for pH sensitivity or structural integrity of the protein; this mutation can cause amelogenesis imperfecta, which alters the structure and appearance of dental enamel [23].

**Figure 1:**
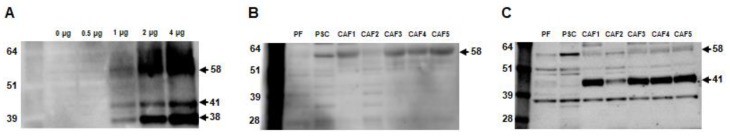
Immunoblotting of GPR68. (**A**) HEK293 cells were transfected with GPR68-v5tag plasmid (0–4 µg). After 48 h, cell lysates were prepared for immunoblotting using V5 antibody (#R960-25, Invitrogen). Three bands were observed, at 58, 41, and 38 kDa. (**B**) Immunoblotting of primary human pancreatic cancer associated fibroblasts (CAFs 1–5), pancreatic fibroblasts (PFs), and pancreatic stellate cells (PSCs) detected GPR68 at 58 kDa. (**C**) PF, PSC, and CAF samples treated with PNGase F (for deglycosylation) shifted the GPR68 band from 58 kDa to 41 kDa.

GPR68 is predicted to have two NH_2_-terminal N-linked glycosylation sites (asparagine-X-serine/threonine (NXS/T) motif, where X is any amino acid) with another putative N-linked glycosylation site in the first extracellular loop [20]. Immunoblotting of GPR68-overexpressing human embryonic kidney (HEK) 293 cells identifies three bands (at 58, 41, and 38 kDa) (Figure 1). Immunoblotting of pancreatic CAFs (which show high expression of GPR68 [24]) detects GPR68 at 58 kDa, which shifts to 41 kDa upon treatment with Peptide-N-Glycosidase F (PNGase F) (Figure 1), implying that GPR68 is glycosylated in cells.

GPR68 can reportedly form a weak homodimer and heterodimers with other GPCRs: GPR4, GPR65, GPR132 and with the lysophosphatidic acid (LPA) receptors, LPAR1 and LPAR2 [25,26]. Chimeric constructs revealed that the N-terminal ‘tail’ of GPR68 is involved in LPAR1-GPR68 dimerization [25]. Heteromerization of GPR68 and GPR132, but not of GPR68 and GPR65, enhanced proton-induced intracellular Ca^2+^ signals [26].

### 2.3. GPR68 Expression in Normal Human Tissues

Northern blot analysis revealed that GPR68 mRNA is expressed in spleen, testis, heart, small intestine and peripheral blood leukocytes (PBL), brain, lung, placenta, and kidney with no detectable expression in thymus, prostate, ovary (even though GPR68 was originally cloned from ovarian cancer cells), colon, liver, skeletal muscle, or pancreas [20]. With respect to specific cell types, GPR68 is expressed in normal human thyroid cells [26], osteoblasts, osteocytes, chondrocytes, epithelial cells of lung, intestine and renal tubules, skeletal myocytes and hepatocytes [22], aortic smooth muscle cells [27,28], airway smooth muscle (ASM) cells [29,30], T cells [31], and neutrophils [32].

**Figure 2:**
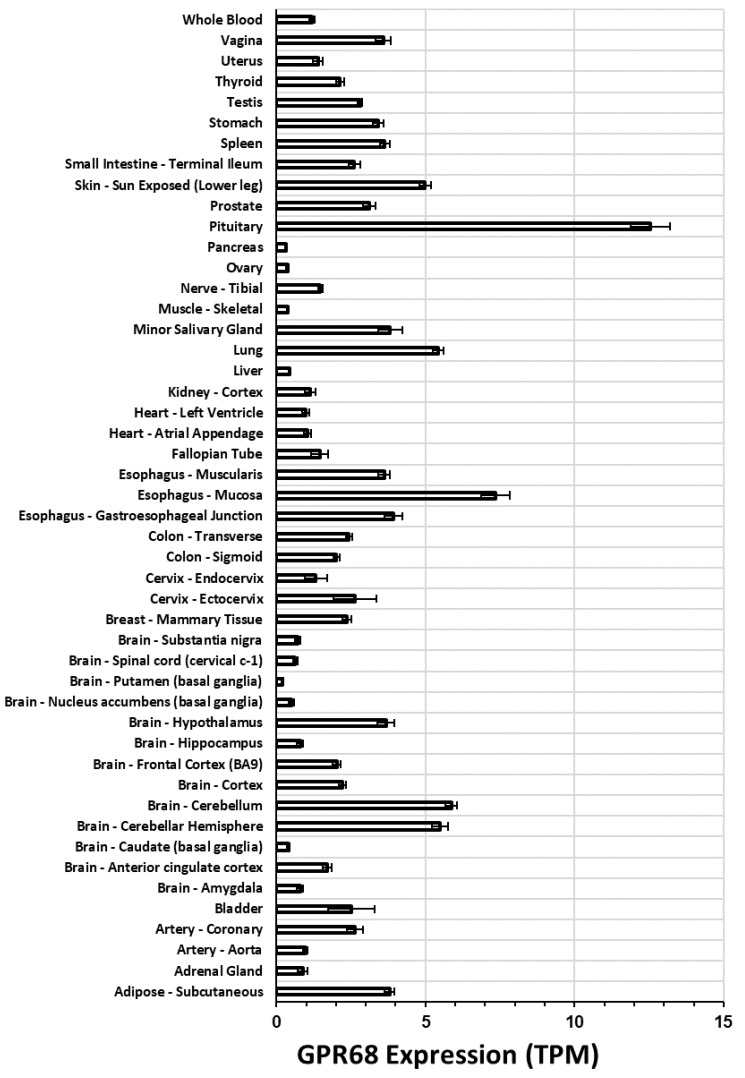
GPR68 expression in human tissues. These data were generated by RNA-seq, quantified in transcripts per million (TPM) from the GTEx database [33], re-analyzed via TOIL [34] and obtained from xena.ucsc.edu. The data shown are average +/− standard error of the mean (SEM) expression in each tissue type.

Based on RNA sequencing (RNA-seq) data from the Genotype-Tissue Expression (GTEx) project (available at xena.ucsc.edu) [33], we analyzed the expression of GPR68 in a range of normal human tissues from data generated via the TOIL pipeline [34] (Figure 2). Consistent with previous data [20], expression of GPR68 is most abundant in the pituitary gland, followed by the esophageal mucosa, cerebellum, and lung. In addition, GPR68 has generally moderate-to-low expression in most human tissues.

### 2.4. Regulation of GPR68 Expression

Factors that regulate GPR68 mRNA expression have been studied. In silico promoter analysis revealed putative DNA-binding sites for activator protein 1 (AP-1), nuclear factor kappa B (NF-κB), and hypoxia inducible factor 1 subunit alpha (HIF-1α) within proximal regions of GPR68 promoter variants [35]. Consistent with those predictions, tumor necrosis factor alpha (TNFα), lipopolysaccharide (LPS), and phorbol myristate acetate (PMA) induced GPR68 expression in primary human monocytes and the MM6 monocytic cell line; NF-κB inhibitors blocked GPR68 expression induced by those treatments [35]. In studies with human pancreatic stellate cells (PSCs) we, too, found that TNFα induces GPR68 expression via the NF-κB pathway [24]. In MM6 cells, hypoxia-enhanced TNFα-induced GPR68 expression was also blocked by inhibition of NF-kB. Chromatin immunoprecipitation analysis revealed that in hypoxia, HIF-1α, but not NF-kB, binds to the GPR68 promoter [36].

High density microarray analysis showed that colony stimulating factor-1 (CSF-1) induced ~7-fold up-regulation of GPR68 mRNA in osteopetrotic rat long bones and receptor activator of NF-κB ligand (RANKL) increased GPR68 mRNA expression >6-fold in mouse bone marrow mononuclear cells [37]. Regulatory factor X6 (RFX6) was identified as a transcription factor that regulates GPR68 expression in a human pancreatic β-cell line, EndoC-βH2 [38]. Promoter region analysis and chromatin immunoprecipitation-sequencing (ChiP-seq) revealed that GPR68 contained binding peaks for Ikaros family zinc finger 1 (IKZF1); IKZF1 functions as a transcriptional repressor of GPR68 in a myelodysplastic syndrome (MDS) cell line [39].

### 2.5. GPR68 Signaling

Activation of GPR68 by extracellular acidity was first shown to stimulate the Gq/11 protein and increase IP formation [22]. The GPR68/Gq/phospholipase C (PLC)/IP3/Ca^2+^ signaling pathway was subsequently reported to regulate connective tissue growth factor (CTGF, also known as CCN2) production in human ASM cells [40]; interleukin (IL)-6 (IL-6) expression in human ASM cells [29]; mucin-5AC secretion in human airway epithelial cells [41]; cyclooxygenase-2 (COX-2) induction and prostaglandin E2 (PGE2) production in human osteoblastic cells [42]; COX-2 expression, prostaglandin I2 (PGI2) production and mitogen-activated protein kinaes-1 (MKP-1) expression in human aortic smooth muscle cells [27,28]; and IL-8 production in the EndoC-βH2 cell line [38].

In addition to Gq-coupled signaling, activation of GPR68 by extracellular acidity also stimulates cyclic AMP (cAMP) accumulation [43,44]. The GPR68/Gs/cAMP pathway was also detected in human aortic smooth muscle cells; it was suggested that cAMP accumulation might occur via GPR68-mediated stimulation of the PLC/COX/PGI2 pathway [28]. In human osteoblastic cells, COX-2-catalyzed PGE2 production is also responsible for pH- and GPR68-dependent cAMP accumulation [45]. However, Mogi et al. [43] showed that neither PLC nor COX inhibitors affect the pH-dependent increase in cAMP accumulation in GPR68-transfected Chinese hamster ovary (CHO) cells, hence implying that GPR68 directly couples to Gs and that pH-dependent cAMP accumulation is not secondary to the Gq-mediated stimulation of Ca^2+^/PLC/COX signaling.

GPR68 signaling pathways have been identified in several human cancers. In pancreatic CAFs, GPR68 activation (by decreasing extracellular pH) enhances IL-6 expression via a Gs/cAMP/protein kinase A (PKA)/cAMP response element-binding (CREB) signaling pathway [24]. Singh et al. [46] reported that constitutive activation of GPR68 in GPR68-overexpressing prostate cancer PC3 cells promoted G protein αi subunit (Gαi) expression and thereby, induced secretion of a migration inhibitory factor. Overexpressing GPR68 in MCF7 breast cancer cells (which endogenously lack GPR68) inhibited cell migration by a Gα12/13-Rho-Ras-related C3 botulinum toxin substrate 1 (Rac1) pathway [47]. No evidence has as-yet been provided that GPR68 acts via β-arrestin signaling.

Huang et al. [48] reported (based on immunostaining of permeabilized cells) that GPR68 was internalized in acidic conditions. Subsequent real-time monitoring of pH-dependent trafficking of GPR68 in living leukocytes showed that GPR68 accumulates on the plasma membrane at mildly acidic extracellular pH (pH 6.6) but is internalized into intracellular compartments by a slightly basic pH jump (pH 7.7) [49]. The contradicting results might be due to different visualization techniques. Further studies are needed to elucidate the internalization pathway of GPR68.

### 2.6. Physiological Roles of GPR68

The generation of GPR68 knockout (KO) mice, first reported by Li et al. in 2009 [50], has helped define functional roles of GPR68. GPR68 KO mice are viable and fertile; histological analyses revealed no significant differences from wild-type (WT) mice in most tissues of the KO mice. Consistent with human data, reverse transcription-polymerase chain reaction (RT-PCR) detected GPR68 in mouse lung, testis, heart, brain, spleen, thymus, brown fat, small intestine, colon, PBL, macrophages, stomach, ovary, and white fat, but not in the liver, kidney, or skeletal muscle [50].

GPR68 has been implicated in regulating osteoclast differentiation [37,51], increasing Ca^2+^ concentration in osteoclasts and enhancing osteoclast survival in acidic conditions [52]. Active osteoblasts express GPR68 [22,42]. By sensing extracellular acidity GPR68 regulates osteoblast differentiation and PGE2 production [42,45,53]. GPR68 KO mice have a decreased number of monocyte CSF (M-CSF)- and RANKL-induced osteoclasts but no observed bone abnormalities [50]. In contrast, Krieger et al. recently reported that bone mineral density and trabecular and cortical bone volume are increased in GPR68 KO mice along with increased numbers of osteoblasts and osteoclasts [54]. Further studies are needed to clarify GPR68’s role in bone formation and resorption.

Nakakura et al. [55] observed that glucose-promoted plasma insulin levels are significantly lower in GPR68 KO mice than WT mice. The authors noted that the GPR68/Gq/11 pathway was activated at pH 7.4 (physiological extracellular pH) and further stimulated by acidification, resulting in enhancement of insulin secretion in response to high glucose concentrations and KCl.

In addition to its ability to sense protons (H^+^), GPR68 was recently shown to be a mechanosensor of shear stress in human and mouse vascular endothelial cells [56] and of stretch in other cell types [57]. Mechanosensing of shear stress required the presence of H^+^ while for mechanosensing of stretch, GPR68 was proposed to be a coincidence detector for membrane stretch and extracellular H^+^ [56,57]. GPR68 KO mice have lower systolic blood pressure than WT mice but without altered diastolic pressure, mean arterial pressure, or heart rate. GPR68 KO mice also have impaired acute flow-mediated dilation and flow-mediated remodeling in mesenteric arterioles [56].

Compared to GPR68^+/+^/IL-10^−/−^ mice (a mouse model of spontaneous colitis), GPR68^−/−^/IL-10^−/−^ mice have a lower incidence and delayed onset and progression of rectal prolapse, implicating a role for GPR68 in promoting experimental inflammatory bowel disease [35]. Such effects may derive from effects of GPR68 on barrier function of intestinal epithelial cells and fibrogenesis in the intestine [58,59].

## 3. GPR68 in Cancer

### 3.1. GPR68 Expression in Cancer

GPR68 somatic mutations are extremely rare in human cancers [3,4]. However, GPR68 is expressed in numerous human tumors, including PDAC tumors and primary PDAC CAFs [24], gastrointestinal stromal tumors (GIST) and appendiceal tumor CAFs [24], colon cancer CAFs [60], anaplastic thyroid cancer (FRO) cells [26], osteosarcoma MG63 cells [22], medulloblastoma tissue [61] and medulloblastoma cell line DAOY [62], Merkel cell carcinoma (MCC), dermatofibrosarcoma protuberans (DFSP), atypical fibroxanthoma (AFX), and pleomorphic dermal sarcoma (PDS) [63].

**Figure 3:**
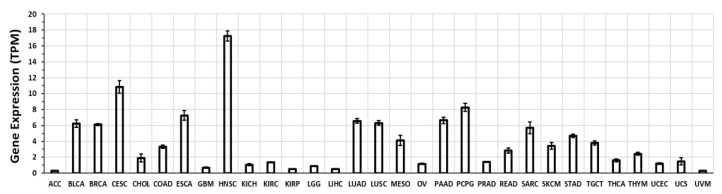
GPR68 expression in solid tumors. These data were generated by RNA-seq, quantified in transcripts per million (TPM) from the Cancer Genome Atlas (TCGA) database [64], re-analyzed via TOIL [34], and obtained from xena.ucsd.edu. Expression indicated are mean and standard error of the mean (SEM) in each tumor type. ACC (adrenocortical carcinoma); BLCA (bladder urothelial carcinoma); BRCA (breast invasive carcinoma); CESC (cervical squamous cell carcinoma and endocervical adenocarcinoma); CHOL (cholangiocarcinoma); COAD (colon adenocarcinoma); ESCA (esophageal carcinoma); GBM (glioblastoma multiforme); HNSC (head and neck squamous cell carcinoma); KICH (kidney chromophobe); KIRC (kidney renal clear cell carcinoma); KIRP (kidney renal papillary cell carcinoma); LGG (brain lower grade glioma); LIHC (liver hepatocellular carcinoma); LUAD (lung adenocarcinoma); LUSC (lung squamous cell carcinoma); MESO (mesothelioma); OV (ovarian serous cystadenocarcinoma); PAAD (pancreatic adenocarcinoma); PCPG (pheochromocytoma and paraganglioma); PRAD (prostate adenocarcinoma); READ (rectum adenocarcinoma); SARC (sarcoma); SKCM (skin cutaneous melanoma); STAD (stomach adenocarcinoma); TGCT (testicular germ cell tumors); THCA (thyroid carcinoma); THYM (thymoma); UCEC (uterine corpus endometrial carcinoma); UCS (uterine carcinosarcoma); UVM (uveal melanoma).

We mined RNA-seq data from the Cancer Genome Atlas (TCGA) (in xena.ucsc.edu) [64], using re-analyzed and normalized data from the TOIL recompute project [34]. Figure 3 shows the level of GPR68 expression in a range of solid tumors. GPR68 is substantially expressed (>5 transcripts per million [TPM]) in several tumor types, notably in head and neck squamous carcinoma (HNSC), cervical squamous cell carcinoma (CESC), pancreatic and lung cancers among others. Thus, GPR68 is expressed in numerous solid tumors.

**Figure 4:**
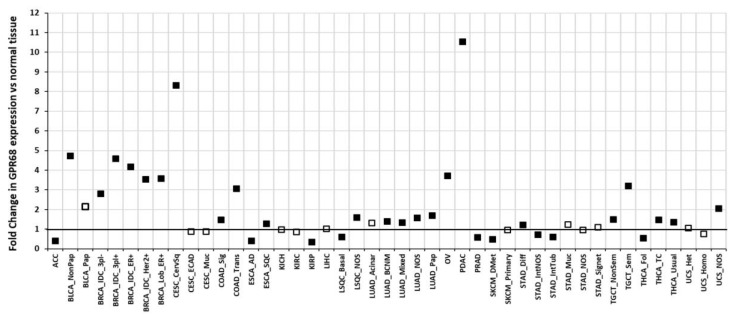
Differential expression of GPR68 in tumors compared to normal tissue. The Cancer Genome Atlas (TCGA) [64] tumors were subdivided into 45 histological subtypes as described previously [24]. Closed squares indicate the fold-changes in GPR68 expression that are statistically significant (false discovery rate (FDR) < 0.05); open squares indicate non-significant fold-changes. Baseline expression in normal tissue is set as 1.

Using RNA-seq data from TCGA (for tumors) and GTEx (for normal tissue) [33], reanalyzed via the TOIL recompute project [34], we performed differential expression (DE) analysis to evaluate DE of GPR68 in solid tumors [24]. Figure 4 shows fold-changes in GPR68 expression for 45 histological tumor subtypes compared to the normal tissues. Of note, 12 tumor subtypes have >2-fold increases in GPR68 expression. The most prominent increases are in PDAC, CESC, and subtypes of breast adenocarcinoma and ovarian cancer, among others. Reduced GPR68 expression occurs in fewer tumor types. The numerous tumors with increase in expression of GPR68 suggest its potential relevance in tumor biology and as a potential therapeutic target in multiple cancers.

**Figure 5:**
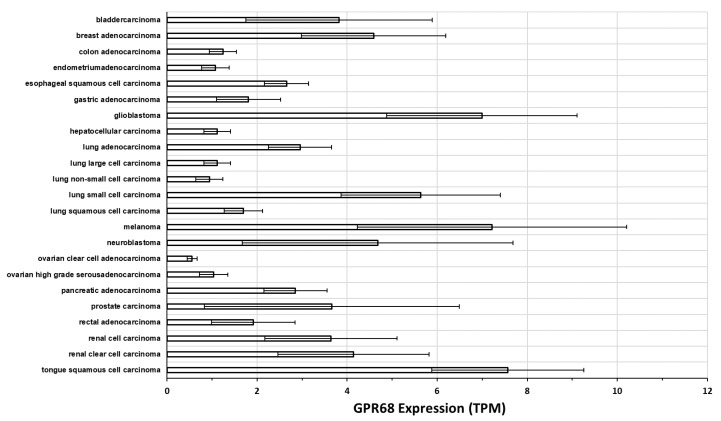
GPR68 expression in cancer cell lines from a range of tumor types. These data were generated by RNA-seq, quantified in transcripts per million (TPM) and analyzed via the iRAP pipeline [66], from the Cancer Cell Line Encyclopedia [65] accessed via the EMBL-EBI expression atlas. GPR68 expression is plotted as mean and standard error of the mean (SEM) across all cell lines.

Figure 5 shows data for GPR68 expression in tumor cell lines from the Cancer Cell line encyclopedia [65], assayed by RNA-seq. These data were obtained from the EMBL-EBI expression atlas (http://www.ebi.ac.uk/gxa), analyzed via the iRAP pipeline [66], generating gene expression in TPM. A number of cancer cell lines show substantial GPR68 expression but several tumor types with elevated expression of GPR68 in tumors (Figure 4) have only modest magnitudes of GPR68 expression in the cancer cells. This raises the possibility that GPR68 may be expressed in other cell types in the TME, as has been shown in pancreatic and colorectal CAFs [24,60].

**Figure 6:**
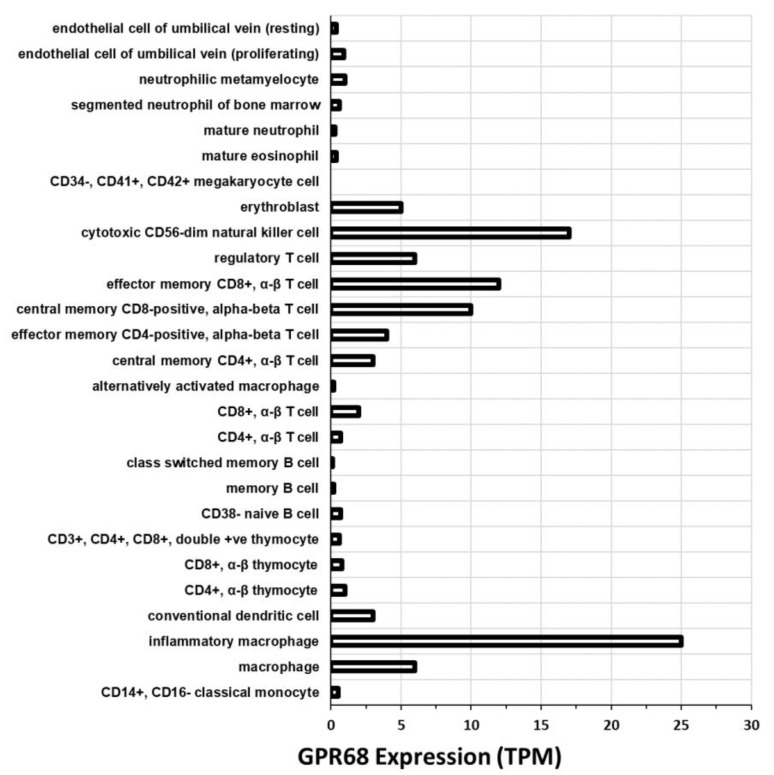
GPR68 expression in hematopoietic cells. These data are from the Blueprint Consortium, hosted at the EMBL-EBI expression atlas, and show GPR68 expression in transcripts per million (TPM) in a variety of cell types.

We also mined data from the Blueprint Consortium (hosted at the EMBL-EBI expression atlas), which provides RNA-seq data for gene expression in hematopoietic cells. The data (Figure 6) indicate substantial expression of GPR68 in numerous immune cell types, particularly macrophages and T cells. Such cells are likely exposed to the acidic TME and may contribute to the expression of GPR68 in solid tumors (Figure 3 and Figure 4). GPR68 signaling may thus impact on immune cell function in tumors. We believe this is an important avenue for investigation.

### 3.2. Biological Functions of GPR68 in Cancer

**Figure 7:**
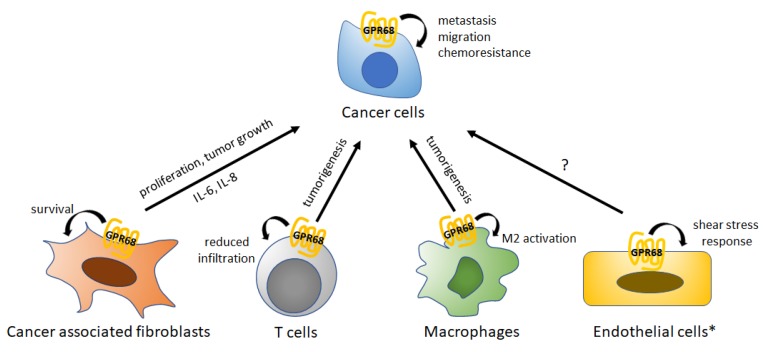
Summary of GPR68 functions in cancer cells and other cells in the tumor microenvironment (TME). GPR68 is expressed on a variety of cells in the TME, each of which has responses to GPR68 activation, as indicated on the figure. * GPR68 has not as-yet been directly shown on tumor endothelial cells, but based on data for vascular endothelial cells are likely to be present in tumors.

In addition to its functions in normal physiology, as noted above, emerging evidence implicates GPR68 as playing key roles in human cancer. Here we summarize such evidence with results for, prostate cancer, melanoma, pancreatic cancer, colon cancer, medulloblastoma, and MDS. GPR68 may be expressed in a variety of cell types in the TME besides tumor cells themselves (Figure 7).

#### 3.2.1. GPR68 in Prostate Cancer

Based on gene expression analysis of prostate cancer, LaTulippe et al. [67] found that GPR68 expression was five-fold lower in metastases than in primary tumors, implying that GPR68 might have an inhibitory role in tumor metastasis. Indeed, Singh et al. [46] reported that mice injected with vector-transfected PC3 (prostate cancer) cells formed tumors in the prostate glands and metastatic tumors in the liver, spleen, kidney, stomach, lung, lymph nodes, diaphragm, and mesentery but mice injected with PC3 cells transfected with GPR68 developed tumors confined to the prostate gland and that did not metastasize. GPR68 did not affect primary tumor growth [46]; consistent with the *in vivo* data, overexpressing GPR68 in prostate cancer cell lines (PC3, C2-4 and DU145) inhibited cell migration, but did not affect cell proliferation. The inhibitory effect was mediated by secretion of a migration factor inhibitor induced by GPR68 in PC3 cells [46]. Similar effects have been observed in GPR68-overexpressing human ovarian cancer HEY cells. Transfection of GPR68 in HEY cells significantly inhibited cell migration and cell proliferation [68]. These data suggest a tumor suppressive role of GPR68 in at least certain prostate and ovarian cancer cells.

Subsequent data indicated that prostate cancer tumorigenesis was reduced in GPR68-deficient mice [69]. Yan et al. [69] showed that TRAMP-C2 prostate cancer cells injected into 23 WT mice resulted in 100% tumor incidence whereas only 7 out of 45 GPR68 KO mice developed tumors. In addition, tumor weights and volumes were reduced in GPR68 KO mice compared to WT mice. Adoptive transfer of differentiation (CD)11b+/Gr1 (Ly-6G/Ly6-C)+ double positive cells isolated from WT, but not from GPR68 KO, mice were necessary and sufficient for tumor development in GPR68 KO mice. Compared to WT mice, GPR68 KO mice have higher expression of an M1 macrophage marker (inducible nitric oxide synthase) and lower expression of an M2 macrophage marker (arginase 1). Furthermore, co-injection of WT macrophages with tumor cells increased TRAMP-C2 tumorigenesis in GPR68 KO mice. Together, these data suggest that GPR68 expression in myeloid lineage cells has a role in tumor cell-induced immunosuppression [69]. 

Yan et al. also showed that GPR68 is expressed at high levels in CD4+ and CD8+ T cells [69]. An increased number of tumor infiltrating T cells was found in subcutaneous prostate tumors from GPR68 KO mice; depleting CD4 or CD8 T cells led to tumor rejection [69]. These findings support the idea that T cells contribute to tumor rejection in GPR68 KO mice. However, adoptive transfer of WT T cells did not significantly affect tumorigenesis in GPR68 KO mice, implying that GPR68 expression in the T cells may not be critical for tumorigenic effects in prostate cancer [69]. Further work is needed to resolve the apparently opposing roles of GPR68 in tumor cells and host cells. 

#### 3.2.2. GPR68 in Melanoma

Li et al. [50] reported that GPR68 deficiency reduced tumorigenesis of melanoma cells. Melanoma B16-F10 cell tumors were significantly smaller in GPR68 KO mice compared to WT mice. Hemotoxylin and eosin (H&E) and CD31 staining revealed fewer blood vessels and CD31+-endothelial cells in the tumors from KO mice compared to those from WT mice, indicating reduced angiogenesis in tumors in KO mice. In addition, fewer epidermal growth factor-like module-containing mucin-like hormone receptor-like 1 (F4/80)-positive macrophages were detected in tumors from the KO mice, implying that decreased tumor-associated macrophages may contribute to reduced tumorigenesis [50]. These data support the hypothesis that GPR68 can have a tumor-promoting role in host cells and the effect is not limited to one tumor type. 

#### 3.2.3. GPR68 in Pancreatic Cancer

In PDAC, GPR68 is highly up-regulated in CAFs compared to CAF precursor cells: PSCs (56-fold increase) and PFs (19-fold increase) [7,24]. Publicly available RNA-seq data reveal that GPR68 expression is 10.5-fold higher in 147 PDAC tumors (TCGA) than in 165 normal pancreas samples (GTEx database). GPR68 expression increases early in tumor development and is highly prevalent in both male and female PDAC patients [7]. As indicated in Figure 5, GPR68 expression is low in pancreatic cancer cell lines and thus its high expression in PDAC tumors (Figure 4) likely reflects expression by cells in the TME other than the cancer cells themselves. The three other members of the proton-sensing GPCR family (GPR4, GPR65, and GPR132) are not detected in pancreatic CAFs [24]. PSCs co-cultured with PDAC cells or incubated with TNFα have induction of GPR68 expression. GPR68 activation (by decreasing extracellular pH) enhances IL-6 expression via a cAMP/PKA/CREB signaling pathway. Knockdown of GPR68 by short interfering RNA (siRNA) diminished low pH-induced IL-6 production and enhancement of PDAC cell proliferation by pancreatic CAF conditioned media. PDAC cells thus induce expression by pancreatic CAFs of GPR68, which senses the acidic microenvironment, thereby increasing production of fibrotic markers and IL-6 and promoting PDAC cell proliferation. Our studies have shown that knockdown of GPR68 expression in CAFs leads to reduced cell viability compared to control cells at acidic condition (pH ≤ 6.6), suggesting that GPR68 provides a survival mechanism for CAFs in acidic conditions [24]. Transfection of GPR68 in PSCs increased expression of fibrotic markers while siRNA knockdown of GPR68 in pancreatic CAFs decreased expression of fibrotic markers; pancreatic CAFs treated with low pH had slightly decreased expression of the pro-fibrotic marker alpha smooth muscle actin (αSMA) and collagen [24]. By contrast, a previous study showed that extracellular acidity promotes transformation of mesenchymal stem cells to CAFs through a GPR68-YAP (yes-associated protein) signaling pathway [70]. The precise mechanism(s) as to how GPR68 regulates fibrotic marker expression in CAFs may vary and requires further study. 

#### 3.2.4. GPR68 in Colon Cancer

Besides its expression in pancreatic CAFs, GPR68 is also expressed in GIST CAFs [24], appendiceal CAFs [24] and colorectal CAFs [60]. Co-culture of human colorectal carcinoma HCT116 cells with human colon fibroblast CCD-18Co cells in 3D spheroid microtumor structures activated CCD-18Co cells to CAFs with a gene signature similar to patient CAFs, among which GPR68 was one of the most highly up-regulated genes during this process. Based on this 3D cancer-cell and fibroblast spheroid co-culture system, Horman et al. conducted a high-content short hairpin RNA (shRNA) genomic screening and identified GPR68 as the number 1 ‘hit’ that regulates microtumor formation [60]. Knockdown of GPR68 in CCD-18Co fibroblasts using virally-delivered shRNAs reduced microtumor spheroid size, IL-6 and IL-8 in the co-culture media. Horman et al. also conducted *in vivo* studies by injection of syngeneic MC-38 murine colorectal carcinoma cells to WT and GPR68 KO mice. During the first 14 days, the tumors grew more slowly in the GPR68 KO than the WT mice and tumors from GPR68 KO animals were more fibrotic, less vascular and had more pronounced borders than tumors from WT mice. After 14 days, tumors in the KO and WT mice had similar growth kinetics; the growth curves were indistinguishable by the end of the study [60]. Overall, the data suggested that GPR68 in host cells promotes colorectal tumor initiation in mice.

#### 3.2.5. GPR68 in Medulloblastoma

GPR68 is highly expressed in human medulloblastoma tissue [61] and medulloblastoma cell line DAOY [61,62]. Huang et al. reported that extracellular acidification activates PLC and subsequent IP3 production, resulting in increased intracellular Ca^2+^ concentration in DAOY cells. The acidification by the PLC/IP3/Ca^2+^ signaling pathway activates the mitogen-activated protein kinase (MEK)/extracellular-signal-regulated kinase (ERK) pathway, which provides a mechanism whereby extracellular acidification may affect cancer cell growth and proliferation [62]. Differentiation of DAOY to less proliferating granule cells did not change GPR68 expression, but in differentiating cells, GPR68 coupled less effectively to PLC activation and IP3 generation, resulting in loss of response to extracellular acidification [71]. GPR68 activation also promoted expression of the proton-potentiated member of the canonical transient receptor potential (TRPC) channel family, TRPC4 and led to opening of TRPC4 channels. TRPC4 can form proton-sensing ion channels and may represent a positive feedback system to promote proton signaling in acidic conditions as activation of TRPC4 channels in DAOY cells resulted in Ca^2+^ influx and enhanced cell migration [61].

#### 3.2.6. GPR68 in Myelodysplastic Syndrome (MDS)

In MDS, a hematological malignancy, genome-wide RNA interference identified GPR68 as a determinant of sensitivity to lenalidomide, a thalidomide derivative used to treat multiple myeloma and MDS [39]. GPR68 knockdown reversed the inhibitory effects of lenalidomide on MDS colony formation and cell viability. Mice transplanted with shGPR68-expressing MDS cells had similar bone marrow engraftment in the lenalidomide-treated and control groups. Lenalidomide treatment degraded the transcription factors IKZF1 and IKZF3, which led to de-repression of GPR68 expression, increased Ca^2+^ concentration and activation of Ca^2+^-dependent calpain, resulting in cytotoxicity in MDS and acute myeloid leukemia cell lines [39].

## 4. Targeting GPR68 with Small Molecules

A family of putative GPR68 agonists, 3,5-disubstituted isoxazoles (Isx), was discovered by functional screening of a library of GPCR-overexpressing cells [72]. Isx compounds activated Gq signaling and increased intracellular Ca^2+^ in GPR68-overexpressing, but not parental, cells. *In vivo*, Isx targeted GPR68-expression in the border zone of infarcted myocardium and drove a pro-survival and cardiomyogenic transcriptional program [72]. Isx can stimulate insulin production [73], promote neurogenesis in young mice *in vivo* [74], inhibit proliferation and induce neuronal gene expression in malignant astrocytes [75]. However the action of Isx seems to be nonspecific [76,77]. Later studies showed that its agonistic action could not be reproduced [44]. 

A yeast-based screen revealed that the benzodiazepine anxiolytic lorazepam is a GPR68 agonist [44]. Based on the GPR68-lorazepam complex model, Huang et al. [44] performed virtual docking of 3.1 million compounds and identified ogerin as a positive allosteric modulator (PAM) for GPR68. Ogerin potentiated low pH-induced cAMP production but inhibited proton-mediated Ca^2+^ release. More recent data [56] indicate that ogerin directly activates GPR68-mediated Gq signaling, suggesting that ogerin is both a PAM for cAMP response and a Gq-agonist for GPR68. *In vivo*, ogerin suppressed recall in fear conditioning in WT, but not GPR68-KO, mice [44]. Different benzodiazepines exhibit preferential activation of GPR68 signaling pathways, with sulazepam selectively activating the canonical Gs signaling pathway in heterologous expression systems, as well as in several primary cell types [67].

Metal ions Zn^2+^ and Cu^2+^ nonspecifically inhibit pH-dependent GPR68 activation by stabilizing the unprotonated state of histidine pairs [22]. A GPR4 antagonist dibenzazepine derivative showed antagonistic effect on GPR68 at high concentration (>1 µM) [78]. Currently there are no selective GPR68 antagonists or negatively allosteric modulators (NAM) although we believe there is a strong need to identify and validate entities that antagonize GPR68.

## 5. Conclusions

GPR68, along with GPR4, GPR65, and GPR132, was discovered as a proton-sensing GPCR that responds to extracellular acidity and alters the function of numerous cell types. Unlike the ligand-receptor binding pocket interaction of other GPCRs [79], GPR68 sensing of H^+^ is thought to occur by protonation of histidine residues, which results in conformational changes and receptor activation. Due to factors that include the challenges of GPR68 as a membrane protein, conformational heterogeneity in solutions and the relatively small structured polar surface available for forming crystal lattice contacts, no crystal structure is as-yet available for GPR68. Perhaps nanobodies that bind and stabilize GPR68 will provide an effective way to solve the crystal structure [79] and can facilitate structure-based drug design. 

Gs and Gq appear to be the primary signaling pathways for GPR68. Cross-talk can occur between these two pathways through COX induction and prostaglandin production in human aortic smooth muscle cells [28] and human osteoblastic cells [45]. In GPR68-expressing model cell lines, GPR68 can directly couple to both Gs and Gq [43]. Perhaps the inconsistent findings derive from different expression levels of GPR68. More cell types need to be tested, in particular cells with endogenous expression of GPR68. Some studies have suggested that GPR68 may also couple to Gi [46] and G_12_ [47]. Such potential signaling pathways need to be explored along with β-arrestin-mediated and signal transduction from intracellular sites. Better understanding of GPR68 signaling may help in design of assays to identify ligands with high potency and low toxicities and provide better insight into the role this receptor plays in normal physiology and disease. 

GPR68 is expressed in multiple tissues and its mRNA expression is increased in numerous tumors (Figure 2, Figure 3 and Figure 4). Emerging evidence implies that GPR68 has key roles in tumor biology. First identified as a metastasis suppressor gene in prostate cancer [23], GPR68 has also been reported to promote tumorigenesis in prostate cancer [69] and melanoma [50]. GPR68 also regulates medulloblastoma cell growth and proliferation [62] and may mediate response to lenalidomide in MDS [39]. CAFs in PDAC, GIST, appendiceal and colorectal cancers also express GPR68; in PDAC, CAF-expressed GPR68 regulates pro-inflammatory cytokine production and promotes tumor growth [24,60]. More studies are needed to assess the role of GPR68 in immune and inflammatory cells in tumors, especially based on its expression in such cells (Figure 6).

Tissue acidity is a hallmark of many pathophysiological conditions, including cancer; it is perhaps not surprising that tumors have evolved to adapt and utilize this altered environment to their advantage. Targeting tumor acidity can suppress tumor growth [17], which further supports the idea that GPR68 is an attractive target for anti-cancer drug development, perhaps as part of combination therapeutic regimens. Until now, few compounds have been identified as selective GPR68 ligands/modulators [44,80] and none are selective GPR68 antagonists with bias toward cAMP signaling. Such compounds, we believe, may be candidates as anti-cancer therapeutics, especially for tumors in which GPR68 enhances their growth or metastatic potential.
